# Risk factors for hepatitis E virus seropositivity in Dutch blood donors

**DOI:** 10.1186/s12879-018-3078-9

**Published:** 2018-04-13

**Authors:** Sofie H. Mooij, Boris M. Hogema, Anna D. Tulen, Wilfrid van Pelt, Eelco Franz, Hans L. Zaaijer, Michel Molier, Agnetha Hofhuis

**Affiliations:** 1 Epidemiology and surveillance unit, Centre for Infectious Disease Control Netherlands, National Institute for Public Health and the Environment (RIVM), Antonie van Leeuwenhoeklaan 9, 3721 MA Bilthoven, the Netherlands; 2 Sanquin Research, Department of Blood-borne Infections, Amsterdam, the Netherlands

**Keywords:** Hepatitis E, Risk factors, Zoonoses, Blood donors, Netherlands

## Abstract

**Background:**

A marked increase of hepatitis E cases has recently been observed in the Netherlands. Causes of the (re-)emergence of hepatitis E virus (HEV) and exact sources and routes of transmission of HEV infection are currently unknown. We aimed to identify risk factors for HEV seropositivity.

**Methods:**

Using the Wantai EIA, 2100 plasma samples of blood donors from all over the Netherlands aged 18-70 years were tested for anti-HEV IgG antibodies. A questionnaire on socio-demographic characteristics, health, and potential risk factors for HEV exposure was sent to these participants.

**Results:**

The overall IgG-seroprevalence was 31% (648/2100) and increased with age. Several food products were independently associated with IgG-seropositivity in a multivariate analysis adjusting for age and gender among 1562 participants who completed the questionnaire: traditional Dutch dry raw sausages called “cervelaat”, “fijnkost”, “salami” and “salametti” which are generally made from raw pork and beef (aOR 1.5; 95%CI 1.2-1.9), frequent consumption of bovine steak (aOR 1.3; 95%CI 1.0-1.7), and frequent consumption of smoked beef (aOR 1.3 95%CI 1.0-1.7). Although not frequently reported, contact with contaminated water was also a risk factor for seropositivity (aOR 2.5; 95%CI 1.5-4.4). Lower seroprevalence was associated with eating raspberries, going out for dinner, and contact with wild animals and dogs.

**Conclusion:**

Several pork food products, mainly dry raw sausages, and contact with contaminated water were associated with past HEV infection in the Netherlands. Further investigation is needed into the prevalence and infectivity of HEV in these risk factor food products, as well as investigation of the production methods and possible origin of HEV-contamination within these sausages, e.g. very small amounts of pork liver, pig-derived blood products as food additive, or the pork muscle tissue.

## Background

Hepatitis E has long been considered a disease mainly affecting developing countries and returning travelers [1]. However, over the last decade it became clear that autochthonous infection with hepatitis E virus (HEV) is common in some industrialized countries, and that in Europe the incidence has increased by ten times [2–4]. The vast majority of HEV-infections in Western Europe are caused by genotype 3, which is known to be a zoonosis with domestic swine, wild boar and deer as the main reservoir [3, 5]. Autochthonous cases may be caused by transmission via food (e.g., consumption of contaminated undercooked meat from pigs, pig-derived blood products used in the food industry, or wild animals) or the environment (e.g., contaminated water or direct contact with infected animals and their feces) [5, 6], but the exact sources and routes of transmission are unknown. Recently Slot et al. demonstrated that the incidence of HEV infection is significantly lower among donors not eating meat compared with meat-eating donors [7]. Moreover, reasons for the current increase of HEV infections in the Netherlands and surrounding countries [4], as observed among young Dutch blood donors [8] and in our national sentinel laboratory surveillance system [9], are currently unclear.

The Netherlands is densely populated by both humans and livestock, including 5.8 million fattening pigs, 1.2 million breeding sows and 5.6 million piglets in 2015 [10]. HEV genotype 3 RNA has been detected in 53% of pig farms, 4% of wild boar feces, and 17% of surface water samples in the Netherlands from 2004 to 2006 [6]. A recent study (2017) showed that residential proximity to pig farms was not associated with increased presence of antibodies against HEV in humans who live in a pig livestock-dense area in the South of the Netherlands [11], which might indicate a primarily food-borne transmission of HEV. Recently in the Netherlands HEV-RNA genotype 3 was detected in a large majority of “liver sausages” (43/55) and “pork liver pâté samples” (12/15) [12], and in 33/36 nonheated liquid pig derived blood products and 7/24 spray dried powder products from pig blood [13]. Other developed countries also reported a broad distribution of HEV-RNA genotype 3 in pork products, among others in liver sausages and dry raw sausages [14–19]. It is currently unclear whether these pork products contained viable and infectious virus, however the level of HEV-RNA-positive samples among common food products is alarmingly high.

Most human infections by HEV genotype 3 are asymptomatic, or present with mild self-limiting symptoms of hepatitis including jaundice, fever, abdominal pain and fatigue. Most symptomatic cases occur among middle-aged and elderly men [1]. In immunocompromised patients there is a risk of chronic infection which can quickly progress to liver cirrhosis, with substantial morbidity and mortality [1]. Therefore, prevention of HEV infection is especially urgent in these high-risk groups. Increased insight in the epidemiology, risk factors and transmission routes of HEV are essential to be able to formulate and implement food regulatory and public health measures for prevention of hepatitis E infection. In this study, we aimed to identify risk factors for HEV seropositivity.

## Methods

### Data collection

This study was performed among blood donors aged 18-70 years who donate plasma at the Dutch blood bank Sanquin. A random selection of 2100 plasma samples collected between March and May 2016 from unique blood donors from all over the Netherlands was tested using an anti-HEV IgG assay (Wantai Biological Pharmacy Enterprise Co., Ltd) according to the manufacturer’s instructions. Borderline reactive samples were considered negative. A detailed, self-administered questionnaire regarding socio-demographic characteristics, health, and potential risk factors for HEV-infection e.g., comprehensive food consumption habits with a focus on meat, contact with (contaminated) water and animals, travel history and outdoor activities was sent to these tested blood donors by post. The recall period used to assess risk factors in the questionnaire was set to 2 months, assuming that food consumption and other habits are relatively constant throughout large periods in life.

Written informed consent was obtained from each participant included in the study. The link between laboratory results, personal identifiers (including postal address) and study number was exclusively retained at Sanquin. Anonymized data were processed and analyzed by the researchers at the National Institute for Public Health and the Environment (RIVM). The ethics committee of Sanquin approved this study, titled “HEV-ID” (acronym for Hepatitis E Virus In Donors).

### Statistical analyses

Associations between determinants and anti-HEV IgG seropositivity (as dichotomous outcome, i.e., yes or no) were assessed using Chi-square tests and univariate logistic regression analyses. Variables associated with seropositivity with a *P*-value < 0.15 in univariate logistic regression analyses were entered into a multivariate logistic regression model. Gender and categories of age were forced into each model, because these were significant confounders of seropositivity being related to many variables that were associated with seropositivity. Subsequently, variables were removed by backward selection to yield a model with the most relevant independent risk factors. With the variables that were included in the multivariate model, additional multivariate logistic regression analyses were performed to assess whether there could be a dose-response effect for food products, by assessing the risk of participants who reported consumption of food products more frequently. Frequency of food consumption was categorized as “often” (several times per week), “regularly” (once per week), “occasionally” (several times per month) and “sometimes” (less than once per month). *P*-values were 2-sided and considered significant at *P* < 0.05. All analyses were performed using Stata software package version 14.

## Results

### Characteristics of the study population and anti-HEV IgG seroprevalence

The overall seroprevalence of anti-HEV IgG was 31% (648 out of 2100 donors) and increased with age. In total, 1562/2100 (74%) donors who were invited into the study completed the questionnaire and could be included in the current analyses. The 538 donors who did not respond to the questionnaire were significantly relatively more often male and significantly younger than the 1562 included participants. Out of 1562 participants who responded to the questionnaire, 1234 (79%) were male and the median age was 58 years (interquartile range 51-64 years); 488 (31%) were anti-HEV IgG-seropositive, and the seroprevalence was highest in males above 60 years of age (39%). There were no significant differences in seropositivity between men and women (32% and 28%, respectively). Seropositivity was similar to results from a previous study by Sanquin among Dutch blood donors [8], in which donations collected in 2011/2012 were analyzed for anti-HEV IgG antibodies using the same assay as in the current study. Nearly all participants reported to be born in the Netherlands (98%) and around half of them reported a high level of education (college or university). In general, the study population consisted of healthy individuals with relatively few health complaints or underlying diseases (Table 1).

**Table 1:** Demographic characteristics of 1562 study participants: blood donors from all over the Netherlands 2016

	Overall (*n* = 1562)		Anti-HEV IgG-negative (*n* = 1074)		Anti-HEV IgG-positive (*n* = 488)	
	N	%	N	%	N	%
Total	1562	100	1074	68.8	488	31.2
*A. Socio-demographic characteristics*						
Gender						
Men	1234	79.0	837	77.9	397	81.4
Women	328	21.0	237	22.1	91	18.7
Median age in years (IQR)	58.1	(50.7-63.9)	56.9	(49.6-63.1)	60.2	(54.5-65.4)
Age groups						
< 30 years of age	24	1.5	20	1.9	4	0.8
30-40 years of age	75	4.8	68	6.3	7	1.4
40-50 years of age	255	16.3	194	18.1	61	12.5
50-60 years of age	561	35.9	392	36.5	169	34.6
> 60 years of age	647	41.4	400	37.2	247	50.6
Country of birth^a^						
The Netherlands	1519	97.8	1041	97.6	478	98.2
Any other country	35	2.3	26	2.4	9	1.8
Level of education^b^						
Low/intermediate	780	50.3	508	47.7	272	56.1
High	770	49.7	557	52.3	213	43.9
*B. Health-related characteristics*						
Previous HEV-test for medical reasons^c^						
No	1368	88.4	927	87.2	441	90.9
Yes	8	0.5	6	0.6	2	0.4
Unknown	172	11.1	130	12.2	42	8.7
Health complaints last 2 months^f^						
Fever	56	3.7	39	3.7	17	3.6
Nausea	54	3.5	41	3.9	13	2.7
Diarrhea < 3 times/day	125	8.2	83	7.9	42	8.8
Stomach ache	91	6.0	67	6.4	24	5.0
Headache	282	18.4	216	20.5	66	13.8
Tiredness	306	20.1	228	21.7	78	16.4
Dark urine	73	4.8	52	5.9	21	4.4
Itching	163	10.6	116	11.0	47	9.8
Neurological symptoms	75	4.9	51	4.9	24	5.0
Underlying diseases last 6 months^f^						
High blood pressure	214	13.7	143	13.3	71	14.5
Diabetes	51	3.3	30	2.8	21	4.3
High cholesterol	126	8.1	82	7.6	44	9.0
Alcohol consumption^d^						
< =6 drinks per week	938	62.5	661	64.2	277	58.7
> 7 drinks per week	564	37.6	369	35.8	195	41.3
Smoking^e^						
No, never	710	46.8	503	48.4	207	43.4
Past smoker	665	43.8	445	42.8	220	46.1
Current smoker	142	9.4	92	8.9	50	10.5
Traveling history (lifetime)						
Asia	687	44.0	479	44.6	208	42.6
Africa	528	33.8	362	33.7	166	34.0
North-America	585	37.5	410	38.2	175	35.9
Central/South-America	417	26.7	306	28.5	111	22.8
*C. Consumption of meat* ^*g*^						
No, never	5	0.3	5	0.5	0	0
No, only in the past	24	1.6	20	1.9	4	0.8
Yes	1518	98.1	1036	97.6	482	99.2

^a^8 missing values

^b^12 missing values

^c^14 missing values

^d^60 missing values

^e^45 missing values

^f^shown if reported by > = 50 participants

^g^15 missing values

### Risk factors for hepatitis E seropositivity

#### Factors related to food, contact with water, animals and lifestyle

In the univariate analyses, age, level of education, lifestyle-related factors like alcohol consumption and going out for dinner, multiple food products including pork products containing pork liver and pork products without pork liver, and factors related to contact with contaminated water or animals, showed a significant association with anti-HEV IgG-seropositivity (Table 2).

**Table 2:** Univariate and multivariate analyses of risk factors associated with anti-HEV IgG-seropositivity among 1562 blood donors. Only variables with *P* < 0.05 in uni- and/or multivariate analyses are shown. The multivariate logistic regression model, adjusted for age and gender, was based on backward selection of variables with *P* < 0.15 in univariate analyses

	Univariate analyses			Multivariate analyses	
	Seropositivity n/N (%)	OR (95% CI)	*P*-value	aOR (95% CI)	*P*-value
*A. General factors*					
Gender			0.121		0.692
Men	397/1234 (32.2%)	1.0		1.0	
Women	91/328 (27.7%)	0.8 (0.6-1.1)		0.9 (0.7-1.3)	
Age groups in years			< 0.001		< 0.001
< 30	4/24 (16.7)	1.0		1.0	
30-40	7/75 (9.3)	0.5 (0.1-1.9)		0.4 (0.1-1.5)	
40-50	61/255 (23.9)	1.6 (0.5-4.8)		1.2 (0.4-3.8)	
50-60	169/561 (30.1)	2.2 (0.7-6.4)		1.7 (0.5-5.1)	
> 60	247/647 (38.2)	3.1 (1.0-9.1)		2.4 (0.8-7.2)	
Level of education			0.002		
Low/intermediate	272/780 (34.9%)	1.0			
High	213/770 (27.7%)	0.7 (0.6-0.9)			
Alcohol consumption			0.042		
< =6 drinks per week	277/938 (29.5%)	1.0			
> 7	195/564 (34.6%)	1.3 (1.0-1.6)			
Traveling history to South America			0.016		
No	377/1145 (32.9%)	1.0			
Yes	111/416 (26.6%)	0.7 (0.6-0.9)			
*B. Food consumption last 2 months*					
*Pork food products*					
Pork tenderloin			0.009		
No	247/867 (28.5%)	1.0			
Yes	241/695 (34.7%)	1.3 (1.1-1.7)			
Pork chop			0.001		
No	311/1083 (28.7%)	1.0			
Yes	177/479 (37.0%)	1.5 (1.2-1.8)			
Pork belly			0.037		
No	267/915 (29.2%)	1.0			
Yes	221/647 (34.2%)	1.3 (1.0-1.6)			
Smoked sausage			0.044		
No	201/702 (28.6%)	1.0			
Yes	287/860 (33.4%)	1.2 (1.0-1.6)			
*Raw or dry sausages*					
Farmer sausage			0.006		
No	340/1159 (29.3%)	1.0			
Yes	148/403 (36.7%)	1.4 (1.1-1.8)			
Traditional Dutch dry raw sausages^a^ (“salami” “salametti” “cervelaat” “fijnkost”)			< 0.001		< 0.001
No	176/673 (26.2%)	1.5		1.0	
Yes	312/889 (35.1%)	1.3 (1.2-1.9)		1.5 (1.2-1.9)	
Liver cheese/ Leberkäse			0.001		
No	371/1267 (29.3%)	1.0			
Yes	117/295 (39.7%)	1.6 (1.2-2.1)			
Pork liver slices			0.001		
No	408/1370 (29.8%)	1.0			
Yes	80/192 (41.7%)	1.7 (1.2-2.3)			
Other dry sausages^e^			0.033		0.022
No	438/1436 (30.5%)	1.0		1.0	
Yes	50/126 (39.7%)	1.5 (1.0-2.2)		1.6 (1.1-2.4)	
*Spreadable pork*					
Regional raw pork sausage “Brabantse metworst”			0.036		
No	471/1526 (30.9%)	1.0			
Yes	17/36 (47.2%)	2.0 (1.0-3.9)			
Tea sausage			0.017		
No	467/1518 (30.8%)	1.0			
Yes	21/44 (47.7%)	2.1 (1.1-3.7)			
Pâté / liver sausage^b^			0.011		
No	197/704 (27.9%)	1.0			
Yes	291/858 (33.9%)	1.3 (1.1-1.6)			
*Beef*					
Steak			0.023		0.022
No	189/671 (28.2%)	1.0		1.0	
Yes	299/891 (33.6%)	1.3 (1.0-1.6)		1.3 (1.0-1.7)	
Smoked beef					0.020
No	318/1107 (28.7%)	1.0	0.001	1.0	
Yes	170/455 (37.4%)	1.5 (1.2-1.9)		1.3 (1.0-1.7)	
Ox sausage			0.021		
No	375/1254 (29.9%)	1.0			
Yes	113/308 (36.7%)	1.4 (1.0-1.8)			
*Fruits and vegetables*					
Raspberries			0.031		0.039
No	429/1328 (32.3%)	1.0		1.0	
Yes	59/234 (25.2%)	0.7 (0.5-1.0)		0.7 (0.5-1.0)	
*C. Food outside the home*					
Restaurant visit			0.003		0.009
< 1 time per month	234/667 (35.1%)	1.0		1.0	
> 1 time per month	234/868 (28.0%)	0.7 (0.6-0.9)		0.7 (0.6-0.9)	
*D. Contact with water or animals in the last 2 months*					
Contact with contaminated water^c^			0.008		0.001
No	458/1497 (30.6%)	1.0		1.0	
Yes	30/65 (46.2%)	1.9 (1.2-3.2)		2.5 (1.5-4.4)	
Contact with dogs			0.066		0.013
No	214/632 (33.9%)	1.0		1.0	
Yes	274/930 (29.5%)	0.8 (0.7-1.0)		0.7 (0.6-0.9)	
Contact with wild animals^d^			0.072		0.025
No	470/1481 (31.7%)	1.0		1.0	
Yes	18/81 (22.2%)	0.6 (0.4-1.0)		0.5 (0.3-0.9)	

aOR: Odds ratio adjusted for age and gender

95% CI: 95%-confidence interval

^a^Combined variable, created based on “salami”, “salametti”, “cervelaat” and “fijnkost”

^b^Combined variable, created based on several (open) questions

^c^Combined variable, created based on “removed water from a stable”, “worked with a septic tank” and “contact with sewage”

^d^Combined variable based on contact with deer (*n* = 7), roe (*n* = 4), wild boar (*n* = 0), hare (*n* = 7), rat (*n* = 9), mice (*n* = 54), and/or other wild animals (*n* = 21)

^e^Variable based on an open question, including very diverse both national and international (local) meat products, five most reported: fuet (*n* = 12, 10.3%), saucisson d’Ardenne (*n* = 10, 8.6%), French dry sausage (*n* = 9, 7.8%), grilled sausage (*n* = 8, 6.9%), smoked bacon called “katenspek” (*n* = 6, 5.2%)

In multivariate analyses, gender and categories of age were forced into each model, because these were significant confounders of seropositivity being related to many variables that were associated with seropositivity. Seropositivity significantly increased with age, with an adjusted odds ratio (aOR) of 2.4 (95% confidence interval (CI) 0.8-7.2) for age category > 60 years compared to < 30 years. Consumption of traditional Dutch dry sausages generally made from raw pork and beef (called “cervelaat”, “fijnkost”, “salami” and “salametti”) was also significantly associated with seropositivity with an aOR of 1.5 (*n* = 889, 95% CI 1.2-1.9). These traditional Dutch dry raw sausages do not contain pork liver. However, consumption of traditional Dutch dry raw sausages was significantly correlated with consumption of pork liver pâté and liver sausages (*P* < 0.001).

Also, consumption of “other dry sausages” showed an aOR of 1.6 (95% CI 1.1-2.4). This was an open question and included very diverse both national and international (local) meat products (fuet (*n* = 12, 10.3%), saucisson d’Ardenne (*n* = 10, 8.6%), French dry sausage (*n* = 9, 7.8%), grilled sausage (*n* = 8, 6.9%) and smoked bacon called “katenspek” (*n* = 6, 5.2%) were most reported). Bovine steak and smoked beef were also significantly associated (*n* = 891; aOR 1.3 95% CI 1.0-1.7) and (*n* = 455; aOR 1.3 95% CI 1.0-1.7), respectively. Furthermore, among 65 persons (4.3% of our subjects) who reported to have had contact with contaminated water (i.e., removed water from a stable, worked with a septic tank or had contact with sewage) we observed increased OR’s (aOR 2.5; 95% CI 1.5-4.4). Interestingly, consumption of raspberries and going out for dinner more than once per month showed a negative association on seropositivity, as well as having had contact with dogs or wild animals in the last 2 months. Consumption of seafood (e.g., mussels, oysters) showed no significant association with anti-HEV IgG seropositivity.

We assessed whether there could be a dose-response effect for food products, using the same variables as included in the previous multivariate model. Within the group of participants who reported consumption of traditional Dutch dry raw sausages, risk significantly increased for participants who reported consumption most frequently as compared to those reported least frequently (*n* = 233; aOR 2.5 95% CI 1.8-3.6) and (*n* = 192; aOR 1.1 95% CI 0.8-1.7), respectively.

#### Health-related factors

None of the health-related factors assessed were significantly associated with anti-HEV IgG-seropositivity in univariate or multivariate analyses adjusting for gender and age and mostly cases per factor were low. In a multivariate model assessing health complaints in the last 2 months only reporting diarrhea less than 3 times per day was borderline significantly associated (*n* = 125; aOR 1.6; 95% CI 1.0-2.5; *P* = 0.052), reporting diarrhea more than 3 times per day was not significantly associated to seropositivity. There were no significant associations between underlying diseases in the last 6 months reported by > 50 participants and seropositivity.

## Discussion

With this study we identified risk factors for past hepatitis E infection that were primarily related to consumption of (pork) food products and contact with contaminated water. The pork food products “traditional Dutch dry raw sausages (called “cervelaat”, “fijnkost”, “salami” and “salametti”)” and “other dry sausages” were independent risk factors for hepatitis E seropositivity. These dry raw sausages are generally eaten unheated, as they are meant to be consumed sliced on bread. While it is currently unknown whether these products, generally made from raw pork and beef, indeed contain (infectious) HEV, transmission of HEV via raw [20] and processed [21] pork products has been suggested before and is biologically plausible. To completely inactivate HEV it is necessary to heat food to an internal temperature of 70° Celsius for at least 20 min [22]. Production and consumption of raw dry sausages like the Dutch “cervelaat”, “fijnkost”, “salami” and “salametti” mainly takes place in Europe. The sausages can vary slightly between different regions and countries. In general, this type of sausages is produced from uncooked, cured pork belly meat mixed with beef. Regularly, the mix of raw meat is fermented, filled in a porous synthetic casing and is hung out to dry until it is solid enough to cut. Remarkably, the pork food products that we found to be risk factors for anti-HEV IgG seropositivity do not contain pork liver tissue. In our study, pork food products that do contain liver (“liver sausages” and “pork liver pâté”) were only independent risk factors in the univariate model, and were no longer significant risk factors in the multivariate model. However, a correlation between the consumption of pork food products that do contain liver and consumption of traditional Dutch dry raw sausages was found. Usually diaphragm tissue is added to Dutch dry raw sausages, which could lead to the presence of very small amounts of liver in the sausages [23].

Besides the liver, HEV is present as well in blood and muscle tissue of pigs during infection with the virus [24, 25]. Pig meat might contain HEV due to the presence of residual blood (1-2%), however the viral dose is probably low due to the small amount of blood. In addition, pig-derived blood products (e.g. spray-dried plasma, red blood cells, globin and collagen) might be used as additives to enhance color and texture of the sausages [26]. Unfortunately, we could not measure the consumption of pig-derived blood products directly, as it is not possible to query about blood products in the questionnaire. Whether HEV transmission might occur following consumption of products containing pig-derived blood products is still unclear. Recently, HEV RNA was detected in 33/36 porcine blood product samples, which are produced for application in the food industry. According to Boxman et al. the liquid blood products are not heat-treated during production, leaving the HEV detected most likely infectious [13]. A study of Szabo et al. detected HEV RNA in 20% of raw sausages that did not contain liver [19]. Furthermore, it might be possible that traditional Dutch dry raw sausages contain residual infectious virus as they do not have a long period of curing, while pork liver sausages are often briefly cooked during processing. It might be possible that although pork products containing liver contain a high amount of HEV RNA, this is associated with heat-inactivated virus particles that are unable to cause infection. Possibly, the level of HEV RNA in the traditional Dutch dry raw sausages is much lower, but associated with viable virus particles.

We found that eating bovine steak and smoked beef were positively associated with seropositivity. However, participants who reported consumption of bovine steak were more likely to report consumption of traditional Dutch dry raw sausages (61.8% versus 50.4%; OR 1.6 95% CI 1.3-2.0), and consumption of “other dry sausages” (10.2% versus 5.2%; OR 2.1 95% CI 1.4-3.1). Also, participants who reported consumption of smoked beef were more likely to report consumption of traditional Dutch dry raw sausages (65.7% versus 53.3%; OR 1.7 95% CI 1.3-2.1).

Participants who reported to have had contact with contaminated water (i.e., removed water from a stable, worked with a septic tank or had contact with sewage) showed relatively high seroprevalence (46%). This is not an unexpected finding, as it is known that in sewage water, surface water and waste water, HEV RNA sequences can be detected that cluster with sequences found in autochthonous cases, swine and wildlife from the same geographical area [5]. Surprisingly, however, we found an independent negative association between anti-HEV IgG-seropositivity and contact with wild animals and dogs, which is in contrast with previous studies [27]. Participants that reported to go out for dinner more than once per month, or to have eaten raspberries in the last 2 months, were less likely to have had past hepatitis E infection, even after adjusting for age and gender. The level of education was significantly higher among participants who reported raspberry consumption and going out for dinner more than monthly, which were negatively associated with anti-HEV IgG-seropositivity. These negative associations with anti-HEV IgG-seropositivity may possibly be due to a higher level of education of these participants and may indicate a healthy lifestyle in general with less exposure to HEV due to for example less meat consumption and less occupations that involve contact with contaminated water. Indeed, a lower level of education was observed among participants who reported contact with contaminated water, which we identified as a risk factor for anti-HEV IgG-seropositivity.

As most infections go unnoticed, serology studies can provide valuable information on prevalence and incidence of HEV infection over different geographical regions and periods in time. In a recent review among European countries the prevalence of anti-HEV IgG antibodies in blood donors ranged from 1.3% (DIAPRO assay, Italy) to 52% (Wantai assay, France) [3]. Among Dutch blood donors seroprevalence was 27% among donations collected in 2011/2012 (Wantai assay) [28]. Our results of the anti-HEV IgG-seropositivity among blood donors in the Netherlands were very similar to those observed 5 years ago [8], i.e. high (31%), unrelated to gender and increasing with age. In this study, we tried to identify risk factors for anti-HEV IgG seropositivity instead of risk factors for acute infection, to identify risk factors associated with past infection and (eating) habits. Moreover, only 0.076% of 59,474 blood donations collected in 2013/2014 in the Netherlands was PCR positive for HEV-RNA [29]. However, in a group of PCR positive donors we would expect recent consumption of a food product with a particularly high risk for HEV, besides the regular (pork) eating habits. For example, in England and Wales consumption of pork pie and consumption of ham and sausages purchased from a major UK supermarket chain was found to be associated with indigenous infection [21].

Strengths of this study include the relatively large number of participants and the use of a detailed questionnaire specifically designed to assess risk factors for hepatitis E infection. A limitation is the discrepancy in timing between the moment of infection and recall period of the questionnaire. The recall period used to assess risk factors in the questionnaire was set on 2 months, thereby assuming that food consumption and other habits are relatively constant throughout large periods in life. The persistence of anti-HEV IgG antibodies after infection still needs further elucidation, as wide population variation in the extent and timing of anti-HEV IgG antibody decay is reported in literature, in which variation between antibody assays probably plays a large role [30]. In addition, the study population consisted of healthy individuals with relatively few health complaints or underlying diseases, and might therefore be not completely representative for the entire population.

## Conclusions

In conclusion, this study identified specific raw pork food products and contact with contaminated water as independent risk factors for past hepatitis E infection. This confirms the general working hypothesis that consumption of pork products is the primary route of transmission in the Netherlands. Raw pork food products that do not contain liver might contain a low amount of infectious HEV, while pork food products that do contain liver might contain high levels of inactivated HEV. There could be some additional transmission of HEV via the environment but our study could show this only in case of intense exposure. Contact with animals seems not to be an important transmission route of HEV for the general population. Further studies are needed to elucidate the role of pig-derived blood products used as additives in food in HEV transmission and it should be determined whether the food products we found as a risk factor indeed contain infectious viral particles. As long as the reasons for the current increase in autochthonous hepatitis E cases in the Netherlands are not understood, it is important for people at risk of chronic hepatitis E infection (e.g., immunocompromised and transplant patients) to avoid eating raw or processed pork products and products that might contain pig-derived blood products.
